# Distinct Episodic Verbal Memory Profiles in Schizophrenia

**DOI:** 10.3390/bs3020192

**Published:** 2013-04-02

**Authors:** Perrine Brazo, Michaelle Ilongo, Sonia Dollfus

**Affiliations:** 1 CHU de Caen, service de Psychiatrie, Caen, F-14000, France; 2 Université de Caen Basse-Normandie, UMR6301 ISTCT, ISTS team, Caen, F-14000 France; E-Mails: dollfus-s@chu-caen.fr (S.D.); michaelle.ilongo@ch-maison-blanche.fr (M.I.)

**Keywords:** schizophrenia, episodic memory, encoding process, storage process, retrieval process, cognitive subtypes

## Abstract

According to some authors, episodic memory impairment may be a feature shared by all schizophrenic patients, whereas others argue in favor of the mnesic heterogeneity. Our aims were to determine whether patients can be grouped based on according to their mnesic performances. The California Verbal Learning Test (CVLT), an episodic verbal learning test, was compared in 61 schizophrenic patients and 61 matched healthy subjects. The 32 indices were calculated using CVLT Scoring Software. This process allowed us to describe patients’ episodic processes in detail (encoding, storage, retrieval). We isolated one group with normative data, another showed impairment of both encoding and retrieval processes, and in the last one, only encoding process was impaired. As schizophrenia is heterogeneous with regard to episodic memory, impairments should not be considered as a common core to the various forms of the illness and it would be fruitful to systematically assess episodic processes in detail to take into account individual abilities and challenges.

## 1. Introduction

Long-term episodic memory underlies the encoding, storage, and retrieval of personal memories and is acquired in a specific spatio-temporal context. In schizophrenia, this prominent cognitive function is considered to be particularly impaired and to constitute a hallmark feature of the illness [1,2,3,4]. Thus impairment of this memory could affect all schizophrenic patients, regardless of the stage or form of the illness [5,6,7,8,9,10,11]. However, authors have showed that some patients can be considered to have a normal memory profile according to their performance on encoding and retrieval tests [12,13]. They have also identified two more mnesic profiles. They showed that the prevailing symptoms, the severity of the disorder, sex, age, age of onset, and duration of illness and treatment varied between groups of schizophrenia patients who were classified according to their memory profiles, emphasizing that episodic memory performances could be heterogeneous in schizophrenia patients.

Furthermore, some reports show that mnesic function correlates with symptom expression in schizophrenia patients, but the evidence has been inconclusive. Thus, positive schizophrenic symptoms seem to be linked to impaired memory [14,15], while some positive schizophrenia patients present with no impairment [16,17,18]. Meta-analyses have concluded that negative symptoms were the only moderator variable significantly linked to memory performances in schizophrenia [1], especially for verbal learning and memory [19].

Using the California Verbal Learning Test (CVLT, [20]), a word list learning task widely used to investigate functional mechanisms of episodic memory, we also showed different episodic memory performances depending on the schizophrenia subtypes. In this study [18], patients with a deficit subtype [21] showed predominant impairment in recall, although their recognition indices were relatively well preserved. Patients with disorganized subtype schizophrenia were characterized by marked impairment to both recall and recognition, whereas the performance of positive subtype schizophrenia patients could not be distinguished from control subjects. However, we were not able to describe precisely how the mnesic processes (encoding, storage, and retrieval) were affected since we analyzed only six CVLT indices. 

Taken together, these data raise the following questions: instead of being a feature shared by all patients with schizophrenia, can memory function identify distinct groups of patients? Can the validity of the grouping be supported by evidence that the groups have distinct clinical characteristics? Therefore, in the current paper, we used all of the CVLT indices to describe episodic processes in detail in order to test the following hypotheses. Episodic memory performances were heterogeneous according to the subjects allowing us to form groups of schizophrenia patients based on distinct memory profiles. Each group had distinct clinical characteristics. 

## 2. Experimental Section

### 2.1. Subjects

Sixty-one schizophrenic were included. The diagnosis of schizophrenia was established using DSM-IV criteria [22]. They were stabilized outpatients with no change to their treatment during the last four months. 

Sixty-one healthy subjects were recruited using advertisements in local newspapers. They had no drug abuse, neurological, or psychotic disorders as assessed by the Diagnostic Interview Schedule (DIS, [23]). 

Patients were matched to healthy volunteers on a subject-by-subject basis with regard to sex, age, and level of education. All participants reported French as their mother tongue. They granted written informed consent. The study was approved by the local ethical committee (CCP of Basse Normandie, France).

### 2.2. Neuropsychological Evaluation

The results of the CVLT allow 32 indices to be calculated to produce a detailed analysis of episodic memory capacity [20,24] (Table 1). It is possible to use the paper/pencil way but the analyses take a long time and we are not able to exclude possible miscalculations. Thus, the appropriate instrument for this purpose was the CVLT Scoring Software which was developed by our research group using Access’97 and based on the first CVLT version. This software allows us to calculate all the indices automatically, except the learning slope (which is calculated in Excel). The Weschler Adult Intelligence Scale (WAIS-R, [25] was used to provide an estimate of Intelligence Quotient (IQ).

**Table 1:** Definitions and calculated values of the norm of the 32 California Verbal Learning Test (CVLT) indices in healthy subjects. Distribution of the indices: Recall [1,2,3,4,5,6,7,8]; Recognition measures [9,10,11]; Recall errors [12,13,14,15]; Recognition errors [16,17,18,19,20]; Organizational strategies [21,22,23,24,25,26]; Contrast measures [27,28,29,30,31,32].

		Definition	Mean (2 SD)
1	List A, trial 1	Measure of initial learning	7.8 (4.5)
2	List A, trial 5	Number of words recalled in trial 5	12.5 (4.8)
3	List A, total recall 1–5	Total number of list A words recalled over five trials	54.5 (19.4)
4	List B, recall	Number of list B words recalled during the immediate recall trial	6.5 (3.7)
5	Short delay free recall	Number of list A words recalled immediately after the list B trial without re-presentation of list A	10.6 (5.9)
6	Short delay cued recall	Number of list A words recalled when category names were provided	11.4 (5.1)
7	Long delay free recall	Number of list A words recalled after a 20-min delay of nonverbal testing	11.3 (5.4)
8	Long delay cued recall	Number of list A words recalled after a 20-min delay of nonverbal testing when category names were provided	11.8 (5.1)
9	Hits	Number of list A words identified during the recognition task that included 28 distractor items	14.6 (2.9)
10	Discriminability	Ability to discriminate targets from distractor items during the recognition task (%)	95.6 (8.1)
11	False positives	Number of distractor items identified as list A items during the recognition task	0.5 (2)
12	Intrusions, total	Total number of nontarget items reported on all free and cued recall trials of lists A and B	2.9 (6.8)
13	Intrusions, free	Total number of nontarget items reported on all free recall trials of lists A and B	2.1 (4.8)
14	Intrusions, cued	Total number of nontarget items reported on the two cued recall trials of list A	0.8 (2.6)
15	Perseverations	Total number of responses repeated on each trial, summed across all free and cued recall trials of lists A and B	4.5 (7.4)
16	List B: shared	List B distractors belonging to a category shared with list A words (fruits, spices)	0.3 (1.2)
17	List B: unshared	List B distractors belonging to a category not shared with list A words (cooking tools, fish)	0.3 (1.4)
18	Prototypic	Distractors that are very common examples of list A categories (e.g., apple)	0.1 (0.8)
19	Phonetic	Distractors with phonetic resemblance to list A words	0.1 (0.5)
20	Unrelated	Distractors without any relation to list A words (e.g., cigarette)	0 (0)
21	Semantic cluster	Ratio of observed to expected clustering in which the proportion of correct responses followed by another correct response from the same category is contrasted with the expected chance clustering	1.8 (0.8)
22	Serial cluster	Ratio of word pairs recalled in the same succession as presented in list A relative to serial ordering expected by chance	2.1 (2.8)
23	Recall consistency	Percentage of target words recalled in one of the first four trials that were recalled in the subsequent trial	0.8 (1.6)
24	Learning slope	Slope of a least-squares regression line calculated to fit changes in correct response scores across trials 1-5	1.1 (1.1)
25	Primacy recall	Percentage of total words recalled in trials 1-5 that were from the primacy region of list A (first four words)	0.3 (0.1)
26	Recency recall	Percentage of total words recalled in trials 1-5 that were from the recency region of list A (last four words)	0.3 (0.1)
27	Retroactive interference	Decrease in recall score between list A trial 5, and list A short delay free recall (%)	−15.6 (34)
28	Proactive interference	Decrease of recall score between list A trial 1, and list B recall (%)	−10.6 (64.2)
29	Storage	Decrease in recall score between list A trial 5, and list A long delay free recall	−1.2 (3.5)
30	Rate of forgetting	Decrease in recall score between list A trial 5, and list A long delay free recall	0.7 (2.8)
31	Improvement index	Recognition hits minus long delay free recall	3.3 (4.6)
32	Improvement rate	Increase in the number of correct responses between long delay free recall and recognition, expressed as a percentage of recall	36.4 (68.7)

### 2.3. Psychiatric Assessment

Patients were evaluated using the Schedule for Deficit Syndrome (SDS, [26]), translated and validated in French [27], and the Positive And Negative Syndrome Scale (PANSS, [28]). The method for defining mutually exclusive symptomatic subtypes was described previously [18]. Residual patients were selected by scores less than 4 on the PANSS positive and negative subscales. Antipsychotic side effects were assessed using the Extrapyramidal Symptom Rating Scale [29].

### 2.4. Data Analysis

#### 2.4.1. Episodic Processes

There is no published norm for the French version of CVLT. Calculations were therefore based on the performance of the comparison control subjects. According to the statistician’s advice, an index score was considered impaired if it was not within two standard deviations of the mean score of control subjects (Table 1). For each patient, we determined which processes were impaired by analyzing all the indices according to the traditional hypothesis whereby an encoding deficit is linked to impaired scores in recall and recognition, and a retrieval deficit is accompanied by a significant reduction in free recall and by a normal recognition score [2]. Accordingly, a patient was considered to have an encoding deficit only if there was a decrease in free recall performance (decrease in indices 1, 2, 3, 4, 5, 7) along with a weak advantage from presentation of cued recall items (6, 8) plus impaired recognition (9, 10). Moreover, the following anomalies were sometimes observed in patients with encoding deficits: the presence of false recognition items of an intrusive nature (11, 18, and/or 19, and/or 20), changes to learning strategies (reduction in index 21 or 23 or 24 and/or increase in index 22 or 25 or 26), failure of proactive interference (28), intrusion recall errors (12, 13 and/or 14), and/or perseveration recall errors (15). 

A patient was considered to have a storage deficit when there was a decrease in the storage index (29), an increase of the rate of forgetting (30), and a failure in retroactive interference (27). 

A patient had a retrieval deficit if the following criteria were met: a decrease in the free recall index (decrease in indices 1, 2, 3, 4, 5, 7) plus an overall improvement in cued recall (6, 8) and in recognition. Patients with retrieval deficits showed normal word recognition and discriminability (9, 10). We observed false recognition (11) of shared (16) and/or unshared categories list B (17). In contrast measures, the index and rate of improvement (31, 32) were increased.

#### 2.4.2. Memory Groups and Clinical Characterization

Patients with similar mnesic function were grouped together. The qualitative variables were compared between the groups using a Chi-squared test or Fisher’s exact test when the validating conditions were not present. Quantitative variables were compared using the Kruskall-Wallis test followed by the Mann-Whitney test to determine significant statistical differences and a Cohen’d to assess the size effects. 

## 3. Results

Patients were strictly matched to healthy volunteers on a subject-by-subject basis with regard to sex (male = 67.2%), age (patients = 35.1 ± 9.9 years old *versus* healthy subjects = 36.1 ± 10.4 years old), and level of education (primary: 4.9%, secondary: 52.5%, A-level (Bac) or above: 42.6%). Total IQ differed significantly between the groups (patients = 87.3 ± 13.4 *versus* healthy subjects = 100.8 ± 10.8, *p* = 0.001).

### 3.1. Memory Groups

Three memory profiles were identified (Table 2). No encoding, storage, or retrieval deficits were observed in 29 out of the 61 patients; this group was therefore considered as having no major memory impairment. Without forgetting that the cut-off score was of 2 SD, we considered this group’s memory activity as “normal” (the normal group). Eleven patients had encoding difficulties without associated storage or retrieval deficits (the encoding group). Finally, 18 patients had encoding difficulties and an associated retrieval deficit (the encoding/retrieval group). Twenty-five patients (41%) showed at least one change in learning strategies (7 patients in the encoding group and all the patients in the encoding/retrieval group). Memory storage alone was impaired in a single patient, and another had storage and retrieval difficulties. We could not define the memory profile for one patient who displayed retrieval difficulties and was impaired in certain encoding indices, but who did not meet all the necessary criteria for deficit of the encoding process.

**Table 2:** Distribution of patients according to memory performance.

Memory performance	Number of patients (*n* = 61)	%
Normal profile	29	47.5
Isolated encoding deficit	11	18
Encoding/retrieval deficit	18	29.5
Isolated storage deficit	1	1.6
Storage/retrieval deficit	1	1.6
Undetermined deficit	1	1.6

### 3.2. Clinical Characterization

Looking at the subtypes, there were 12 deficit, 9 disorganized, 19 positive, and 21 residual patients. The patient with isolated storage difficulties was a deficit patient. The patient with storage and retrieval difficulties and the patient with the undetermined profile had residual schizophrenia. They were excluded from the remainder of the study. 

Patients’socio-demographic and clinical characteristics are summarized in Table 3. All patients were treated with antipsychotics (classical or atypical antipsychotics such as clozapine, olanzapine, and risperidone). Distribution of gender (*p* < 0.05), subtypes (*p* = 0.005) and level of education (*p* < 0.05) differed significantly between the groups. The normal group encompassed most of the females and most of the residual patients. The encoding/retrieval group was characterized by the worst level of education. All the disorganized patients had mnesic impairment and the detail of the results showed that they had, at a minimum, a change in learning strategies. 

Some quantitative variables differed significantly across the groups. Post-hoc tests and Cohen’s d were carried out. The age of onset (z = −2.7, *p* = 0.007, d = 0.77) and IQ (z = −3.47, *p* = 0.0005, d = 1.37) were greater in the normal group than in the encoding/retrieval group. Compared to the normal group, the Parkinson scores [29] were higher in the encoding/retrieval (z = −2.17, *p* = 0.03, d = 0.74) and encoding (z = −2.04, *p* = 0.04, d = 0.55) groups. 

**Table 3:** Clinical and demographic characteristics of schizophrenia patients grouped according to mnesic profile.

Group	Normal	Encoding	Encoding/ retrieval	*p*
	*n* = 29^a^	*n* = 11^a^	*n* = 18^a^	
Age (y)	35.3 (10.9)	35.4 (11)	35.3 (8.2)	ns *
Age of onset (y)	24.8 (5.5)	22.2 (5.2)	20.8 (4.8)	< 0.05 * H = 7.88
Duration of illness (y)	9.3 (9.5)	9.7 (7.5)	12.1 (7.6)	ns *
PANSS positive subscale	11.2 (4.2)	13.3 (5.4)	12.8 (3.9)	ns *
PANSS negative subscale	14.4 (5.2)	15.5 (5.9)	17.4 (7.2)	ns *
PANSS total score	52.2 (12.3)	57.1 (15.5)	61.1 (13.5)	ns *
Total IQ	92.4 (13.5)	86.2 (9.6)	76.1 (10.1)	0.001 * (H = 3.21)
Subtypes				0.005 **
- Positive	10 (34.5%)	4 (36.4%)	5 (27.8%)	
- Deficit	5 (17.2%)	1 (9.1%)	5 (27.8%)	
- Disorganized	0 (0%)	3 (27.3%)	6 (33.3%)	
- Residual	14 (48.3%)	3 (27.3%)	2 (11.1%)	
Male	55.2%	72.7%	88.9%	< 0.05 **
Level of education				< 0.05 **
- Primary	0%	9.1%	11.1%	
- Secondary	44.8%	45.5%	72.2%	
- A-level (Bac) or above	55.2%	45.4%	16.7%	
Atypical antipsychotics	48.3%	20%	27.8%	ns **
Anticholinergics	24.1%	27.3%	50%	ns **
Parkinsonism score	6 (6.8)	10.1 (8)	12.3 (10)	< 0.05 * (H = 6.86)

Notes: ^a^ mean (standard deviation) or number (%); * Kruskall-Wallis test (degrees of freedom = 2); ** Fischer test; PANSS (Positive and Negative Syndrome Scale); IQ (Intelligence Quotient).

## 4. Discussion

Using all the CVLT indices, we described the episodic memory processes of 61 schizophrenia patients in detail. To the best of our knowledge, no study has used all the CVLT indices to analyze schizophrenic patients’ memory abilities. For example, Paulsen *et al.* [12] or Turetsky *et al.* [13] solely used a part of the indices of the CVLT.

Three distinct CVLT performance/memory profiles were isolated, *i.e.* the normal, encoding, and encoding/retrieval groups (Table 2). They characterized 95% of the patients. We identified clinical characteristics for each group (Table 3). However, they differed in terms of mean scores and no variable appeared to have sufficient predictive value to indicate if a patient belonged to one of the memory group. 

### 4.1. Heterogeneity in Episodic Memory Function

Our results do not support the hypothesis that episodic memory impairment is a hallmark feature of schizophrenia which is common to the various forms of the illness [3,4,8,9,10]. They are in agreement with studies which report that schizophrenia patients have distinct memory profiles. Some of them may be characterized by no major memory impairment: 47.5% of the patients in this study (the normal group); 35% for Paulsen *et al.* [12]; 51% for Turetsky *et al.* [13]; 40% for Brazo *et al.* [18]; and 33% or 39% for McDermid Vaz and Heinrichs, depending on the grouping method [30]. Weickert *et al.* [31] based their study on a clinically derived subgroups approach. They concluded that 25% of their population (117 patients with schizophrenia) were “intellectually intact” compared with healthy subjects, the neuropsychological evaluations including some CVLT scores. They later performed on the same population “atheoretical” cluster analyses which allowed to validate these results.

As for the episodic processes, the hypothesis that memory storage shows subtle or relatively rare impairment [1,2,5,6,12,13,32,33,34,35] was supported by our results since only two patients had a storage deficit. In our study a small group showed only an encoding deficit. This is in agreement with some authors who noted that an isolated encoding deficit is possible [32,33]. 

### 4.2. Clinical Characterization of Memory Groups

Some clinical characteristics differed significantly between the normal and encoding/retrieval groups. The encoding group had characteristics that were intermediate between the other two groups. 

Most of the women fell into the normal group, while the encoding/retrieval group was made up almost exclusively of men. Women usually perform better on the CVLT than men [20,36]. This has been confirmed in patients with schizophrenia [37,38,39] as well as among their first-degree relatives [40]. Moreover, women traditionally have a later age of onset than men, which might explain the difference in age of onset between the normal and the encoding/retrieval groups (*p* = 0.007).

IQ and level of education were higher in patients in the normal group than in patients in the encoding/retrieval group which had the lowest IQ and level of education. The influence of IQ on CVLT results is well documented. Certain WAIS-R tests [25] require memory skills, which could also explain these differences. Yet Hill *et al.* [6], along with Holthausen *et al.* [33], McDermid Vaz and Heinrichs [41] and Paul *et al*. [42], showed that there is evidence of episodic impairment, even when IQ and level of education are taken into account. Cirillo and Seidman [2] reached the same conclusion in their review article which encompassed 19 studies addressing the relationship between intelligence and memory test performance. In our results, we emphasized that IQ performances did not differ significantly between the normal and the encoding groups on the one hand, and between the encoding and the encoding/retrieval groups on the other hand.

Our results suggested that the extent of mnesic deficit might vary according to symptomatic subtype but we failed to isolate only one memory profile for each subtype. Overall positive subtype performed better than disorganized and deficit subtypes, confirming previous findings [18,43]. However, 26.3% of positive subtype had encoding/retrieval deficits. This might explain why positive patients are considered normal by some [16,17,18] and impaired by others [14,15]. 

Like Turetsky *et al.* [13], we found that the deficit patients had one of three memory profiles; half of them were in the normal group. In agreement with other reports [44,45,46,47,48], we therefore postulate that mnesic function does not allow us to distinguish these patients from nondeficit schizophrenic patients. 

On the other hand, patients with the disorganized subtype could be distinguished by the gravity of their cognitive difficulties, and by their mnesic problems in particular [13,43]. Indeed, all these patients without exception had memory impairment (with the alteration of at least one learning strategy).

Regarding the residual patients, 73.7% performed normally on the CVLT. However symptom severity did not seem to be a major influential factor [1,2]. Indeed, the normal group consisted of patients that were heterogeneous in terms of symptom level: half of them were residual patients with low symptom intensity, whereas the other half were positive and deficit patients with more severe symptoms. Moreover the total PANSS scores of the three memory profile groups did not differ (Table 3). This is consistent with studies that have emphasized the tenuous or absent connection between symptoms and mnesic impairment, either in terms of severity [1,6,33,34,49] or in terms of evolution over time [2,50]. 

### 4.3. Practical Outcomes

What can we highlight from these data? Taking into account the clinical aspects, our results sustain that episodic memory efficiency may differ according to some characteristics of schizophrenia. Indeed, the normal group was characterized by congruent clinical characteristics evocative of a moderate form of illness (the majority of residual patients, the majority of the women, a late age of onset, a high average IQ, a high average level of education, low Parkinson scores). In contrast, the group of patients with the most severe mnesic dysfunction (the encoding/retrieval group) had clinical characteristics that tended towards a more debilitating form of illness (the majority of disorganized patients, mostly male, an early age of onset, a low average IQ, a low average level of education, high Parkinson scores). Thus, these results suggest it would be fruitful to systematically assess episodic processes in detail to take into account individual abilities and challenges.

Focusing on mnesic processes, we emphasize that memory storage capacity was, on the whole, intact. Even for the patients with the weakest scores on the CVLT, the problem was access to vocabulary rather than memory destruction per se. It might be helpful to use cognitive remediation therapy that focuses on encoding and retrieval strategies. Some studies have found that encoding is predominantly impaired. Superficial encoding (like serial encoding) is used more often by patients [51], but it is used less effectively than in healthy subjects [32,52]. Recourse to a semantic encoding strategy (also called deep encoding) is often a predictor of verbal learning capacity. Patients use this strategy less often or less effectively than healthy subjects do [5,6,12,32,34,35,42,52]. However, forced use of deep encoding strategies can help patients improve [4,5,32,42,52,53,54]. Thus, it might be helpful to teach remediation techniques that concentrate on practicing the choice of these semantic encoding strategies [51,55].

### 4.4. Limitations

This study has some limitations. First, we studied stabilized patients. Therefore, interpretation of the results should be limited to non-acute schizophrenia patients. On the other hand, patient status could not affect the magnitude of memory impairment [1].

Second, we did not take into account medications as confounding variables. A high proportion of patients in the normal group were taking atypical antipsychotics; this group had a low percentage of patients with anticholinergic prescriptions. In contrast, a high proportion of patients in the encoding/retrieval group were taking standard neuroleptics; this group had a high percentage of patients with anticholinergic prescriptions. On the one hand, Cirillo and Seidman [2] concluded that standard neuroleptics had very little effect on memory abilities whereas atypical medication could improve performances; on the other hand, Keefe *et al.* [56] emphasized that antipsychotics improve memory performance slightly, with no major gain compared to standard neuroleptics. About anticholinergics, they are generally thought to have a negative impact on memory, particularly on semantic encoding [57]. However, the studies we took into account in this paper concluded that this effect was probably negligible [12,41] or nonexistent [30]. Cirillo and Seidman’s conclusions reinforced this point of view [2]. In sum, although medication influence should not be neglected, this effect cannot solely account for the cognitive group differences.

A third limitation concerned cognitive aspects. We should have specifically studied covariables such as executive function and attention. Because processes like categorizing (executive function) or extracting specific information from a given context by suppressing irrelevant information (selective attention) are required during semantic and serial regrouping mechanisms which play a role in the encoding of CVLT items [58]. Moreover, when evaluations have been provided, WAIS-R was the only version available in France. The use of an outdated version of the WAIS can have induced a risk of Flynn effect. We cannot exclude that the IQ measured with our norms would be lower than IQs measured with updated WAIS norms. 

Finally, the validity of grouping schizophrenic patients using mnesic function needs to be tested further using longitudinal evaluation [30]. 

## 5. Conclusions

Our results confirm that schizophrenia is heterogeneous with regard to episodic memory. First, distinct groups of patients were identified on the basis of memory function. The groups differed in terms of mean scores on numerous clinical variables. However, we failed to highlight specific features in each group. So it remains unclear whether episodic heterogeneity fits into distinct memory subtypes or into different degrees of illness severity. We think it would be relevant to include criteria other than clinical variables to support the validity of the mnesic groups and to clarify this question. Second, as impairments should not be considered as a common core to the various forms of the illness, episodic processes should systematically be assessed in detail to take into account individual abilities and challenges. 
